# Validation of a Computerized Version of the Trait Subscale of the Physical Appearance State and Trait Anxiety Scale in Mexican Preadolescents

**DOI:** 10.3390/children9010064

**Published:** 2022-01-05

**Authors:** Juan Francisco Aguirre, Yunuen Socorro Rangel-Ledezma, Perla Jannet Jurado-García, Humberto Blanco, Martha Ornelas, Carolina Jiménez-Lira, José René Blanco, Susana Ivonne Aguirre

**Affiliations:** Faculty of Physical Culture Sciences, Autonomous University of Chihuahua, Chihuahua 31000, Mexico; jaguirre@uach.mx (J.F.A.); yrangel@uach.mx (Y.S.R.-L.); pjurado@uach.mx (P.J.J.-G.); hblanco@uach.mx (H.B.); mornelas@uach.mx (M.O.); cajimenez@uach.mx (C.J.-L.); jblanco@uach.mx (J.R.B.)

**Keywords:** validity, reliability, psychometry, psychological evaluation, anxiety, kids, schoolchildren

## Abstract

Anxiety is a feeling of fear, dread or restlessness and can develop into a weight-related disorder. The objective was to analyze the psychometric properties of the trait anxiety subscale of the Physical Appearance State and Trait Anxiety Scale (PASTAS), as well as the invariance in Mexican preadolescents. The sample consisted of 604 participants, 285 female and 319 male, whose ages ranged between 11 and 12 years (M = 11.37; SD = 0.48). The questionnaire’s factor structure was analyzed using confirmatory factor analyses. The analyses show the viability and adequacy of a two-factor structure (weight and non-weight factors) both for the total sample and for the populations of male and female. The two-factor structure showed adequate reliability and validity fit indicators. The factor structure, the factor loadings and intercepts are considered invariant according to the variable sex; however, differences between female and male participants were found for levels of anxiety caused by physical appearance. In conclusion, the PASTAS can be considered a convenient instrument to assess the variables related to anxiety generated by one’s physical appearance, as well as allowing more participants to be quickly assessed.

## 1. Introduction

Anxiety is a feeling of apprehension or fear in the face of an adverse situation and manifests itself through transitory physiological and psychological reactions at a specific time that enable the person’s ability to respond [1] 

It is convenient to underscore the importance of differentiating between anxiety as an emotional state and anxiety as a personality trait. Anxiety as a personality trait describes the relatively stable individual differences in anxiety, related to a tendency, disposition to the current state of anxiety. This anxiety is not directly manifested in behavior and must be inferred from the frequency of increased anxiety experienced by the individual; whereas, anxiety as an emotional state is an immediate emotional state, modifiable over time, characterized by a unique combination of feelings of tension, nervousness, worry, apprehension and annoying thoughts together with physiological changes. In this sense, individuals with a high degree of trait anxiety perceive a greater range of situations and contexts as threatening and are more predisposed to suffering more frequently or presenting greater intensity of anxiety [2,3], becoming more susceptible to stress [4].

Anxiety is common during the preadolescence and adolescence stage and has been associated to low academic performance, school failure, the quality of the interactions in the school, within the family and social other circles [5,6]. In addition, during this stage of life, anxiety is associated with various psychological disorders such as body dissatisfaction, depression, weight gain and obesity. In this way, preadolescents’ and adolescents’ anxiety can be directed towards recurring thoughts and a constant concern with body image [7].

An additional source of pressure during this period of transition and psychosocial adaptation may be related to the media. The constant projection an ideal standard of beauty may, increase the discrepancy between the ideal physique and the body itself and affect the development of a healthy and coherent sense of self [8]. This influences preadolescents’ and adolescents’ autonomy and future problem solving abilities, causing concerns about self-concept, body weight, self-esteem, body dissatisfaction and eating disorders; reinforcing behaviors that result in weight gain for both men and women [9,10]. 

Some studies related to anxiety in conjunction with obesity and depression indicate that people with obesity show a greater risk of gaining weight due to increased appetite, which is a symptom that is frequently present in anxiety and mood disorders. A negative correlation has been found between trait anxiety and self-efficacy to lose weight, as well as between trait anxiety and loss of body weight after a twenty-week nutritional treatment [11,12,13,14]. 

Overweight adolescents present a greater susceptibility to self-devaluation of body image [15], leading them to feel down, have low self-esteem, unconformity with their body, suicidal ideations, anxiety, depression and eating disorders [16]. In the health field, previous studies have analyzed the link between body dissatisfaction, body image, weight and anxiety in different countries and different age groups [17,18,19], reporting a strong correlation between them, and such studies have allowed the development of therapeutic strategies [20], validation and adaptation of measurement instruments related to anxiety and weight [21] that contribute to the search for balance in the development of individuals.

In Mexico, results from the National Health Survey for School-aged Children (ENSE 2010) [22] reported various inappropriate eating behaviors in 10-year-old preadolescents. Ten percent of the schoolchildren reported at least one inappropriate eating behavior, such as self-induced vomiting practices, restrictive practices such as fasting, dieting or excessive exercise with the intention of losing weight; others reported a feeling being unable to stop eating. Results by sex indicated that male primary school students who practiced restrictive practices were more (3%) than women of that same educational level (2%).

With regards to body image perception and gender differences, Ornelas and collaborators in 2018 [23], in a sample of students with a mean age of 12.95 years, reported that boys have lower levels of anxiety and higher self-esteem than girls, who, in relation to boys, show greater dissatisfaction with their body image, reflecting the desire and perceived need to have a slimmer body.

Anxiety levels have also been found to vary with age, for example, Hernández et al. (2018) [6] reported in a study with students whose ages ranged between 12 and 16 years, that, as the students become older, the probability of increasing their levels of state anxiety also increases and more so the levels of trait anxiety. They also indicated that there is a negative relationship between self-esteem and emotional state anxiety and trait anxiety. 

Given the findings from the literature and that this instrumental study [24] has the goals of analyzing (a) the psychometric properties of the Physical Appearance State and Trait Anxiety Scale (PASTAS)) by Reed, Thompson, Brannick and Sacco (1991) as well as (b) the factorial invariance by sex in Mexican preadolescents, two hypotheses were formulated: The first is that there will be a two-factor structure of the trait subscale of the PASTAS questionnaire (i.e., the weight-related and the not weight related factors); second, that the structure will be invariant in male and female Mexican preadolescents.

## 2. Materials and Methods

### 2.1. Participants

Six-hundred-and-four preadolescents from northern Mexico, fifth (298) and sixth year (306) of primary school, 285 female and 319 male (Table 1) participated in the present study. The sample was obtained through convenience sampling. Participants’ mean age was 11.37 years (SD = 0.48, range = 11–12 years).

### 2.2. Instrument

Physical Appearance State and Trait Anxiety Scale (PASTAS) For the present study, only the trait subscale was used. The original questionnaire consists of 16 items that assesses trait-anxiety associated with body weight (8 items: Body weight, Thighs, Buttocks, Hips, Abdomen, Legs, Waist and Muscular tone) and areas of the body that are unrelated to weight (8 items: Ears, Lips, Wrists, Hands, Forehead, Neck, Chin and Feet), where the participant responds, to the question: In general, how anxious, tense or nervous do I feel about: each one of the 16 areas or body parts previously mentioned in relation to weight and not related to weight.

For our study, in addition to translating the items of the questionnaire, two adaptations were made to the version of Reed and collaborators [25]. For this reason we will refer to the PASTAS questionnaire as the PASTAS-M questionnaire (M for modified)

First, in contrast to the version developed by Reed et al., which provides the participant with five-answer options, the version used in the present investigation, presents the participant with 11 possible answers to express his level of anxiety. The combination of the original scale and our version resulted in the following response options: not at all (0), slightly (1, 2 and 3), moderately (4, 5 and 6), a lot (7, 8 and 9) and too much (10) This modification was made because the participants are students who are accustomed to being graded on a scale from 0 to 10 by the educational system of Mexico.

Second, the questionnaire was completed on a computer; this was done to facilitate data storage, eliminating the need for prior coding, making the process faster and more precise.

It is important to note that the PASTAS questionnaire has been studied in adolescents and young Mexicans, but not within the age range of the sample included in our study (i.e., preadolescents) and therefore, the intention to continue using it to provide data that will enable future comparisons by age group [26].

### 2.3. Procedure

The research protocol has been approved by the Scientific Committee of the Department of Research and Postgraduate Studies at the Faculty of Physical Culture Sciences of the Autonomous University of Chihuahua. In addition, this research met the guidelines of the regulations of the Mexican General Health Law on Research for Health. For informed consent, contact was made with the educational authorities who were in charge of speaking with the parents through the director of each institution. Once the permits were obtained, elementary students from the city of Chihuahua, Chihuahua, Mexico were asked whether they would like to take part in the study. Students completed the questionnaire by means of a computer, and before accessing the instrument, informed consent was had to be granted. To sign, the participant had to press the “Yes, I agree” button; if the “I don’t agree” button was pressed, the system immediately abandoned the questionnaire. It was also made clear to the students that at any time they did not want to continue filling in the questionnaire, they could abandon it. The instruments described above were then completed on a personal computer (administrator module of the instrument of the editor of typical execution scales), in a single, approximately 40-min session, in the classrooms of the educational centers. Participants were given a brief introduction prior to accessing the instrument, about the relevance of the study and instructions on the process of accessing the instrument. The first screens (before item 1 was shown) included instructions on how to respond. Once the participant finalized his or her participation, he or she was thanked by a member of the research team.

Upon completion of the questionnaire, the data were collected and analyzed using the JASP version 0.14.1 package [27].

### 2.4. Data Analyses

The psychometric analyses were carried out in two stages: (1) Analyses of the psychometric properties of the instrument and (2) factorial invariance analyses to obtain a test that presents the best psychometric properties.

#### 2.4.1. Analysis of the Psychometric Properties of the Instrument

Values of skew and kurtosis for each item were calculated as an initial step, to determine if the normality assumption was met for the analyses of the psychometric properties of the questionnaire.

The next step was to perform a comparison of two measurement models: the PASTAS-M16, which corresponds to a two-factor structure with the original placement of the items within the questionnaire, and the PASTAS-M10, which has the factor structure of the PASTAS M-16 but for which inadequate items were removed, as suggested by the modification indices.

Finally, Cronbach’s Alpha Coefficient [28,29] and the Omega Coefficient [30] were calculated to assess factor reliability for the best of the measurement models obtained.

#### 2.4.2. Analyses of Factorial Invariance

In order to obtain a test that presents the best properties for the conformation of the scores of the PASTAS-M questionnaire in females and males, an analysis of factorial invariance was performed; the analysis was performed on the best model obtained from the total sample (PASTAS-M10 model). Cronbach’s Alpha and the Omega Coefficient [30] were calculated to assess the reliability of each dimension for both samples. 

To conduct all the confirmatory factor analyses, the JASP version 0.14.1 [27] software was used, the variances of the error terms were specified as free parameters. In the latent variables (factors) one of the structural coefficients was set to one, so that its scale is equal to that of one of the observable variables (items). The estimation method used was the Maximum Likelihood (ML) with the application of bootstrap resampling procedures for cases of non-normality [31,32]—even though the lavaan package [33] is especially robust for possible cases of non-normality, in large enough samples, and the values of skew and kurtosis are not extreme (skew < |2| and kurtosis < |7|)—our analytic procedure followed Thompson (2004) [34] and not only corroborated the fit of a theoretical model, but also compared the fit indices of alternative ones.

To assess the fit of the model, the Chi-squared statistic, the goodness-of-fit index (GFI) and the mean square error of approximation (RMSEA) were used as absolute measures of fit. The Adjusted Goodness Index (AGFI), the Tucker–Lewis Index (TLI) and the Comparative Goodness-of-Fit Index (CFI) were applied as measures of incremental fit. The Chi-squared over degrees of freedom ratio (CMIN/GL) and the Akaike Information Criterion (AIC) were used as parsimony fit measures [31,35].

## 3. Results

### 3.1. Skew and Kurtosis Values of the Questionnaire Items

Table 2 presents the means, standard deviations, values of skew and kurtosis for the items considered in the measurement model. Most of the variables show skew values between −1.5 and +1.5 and −2.0 to +2.0 for kurtosis, thus, the variables are reasonably normally distributed; on the other hand, the Mardia multivariate index above the value of 70 indicates absence of multivariate normality [36].

### 3.2. Confirmatory Factor Analysis of Total Sample

The overall results of the CFA (GFI 0.901; RMSEA 0.081; CFI 0.939) for the PASTAS-M16 model, which corresponds to a two-factor structure, indicate an acceptable measurement model (Table 3).

The factors of the PASTAS-M16 model explain approximately 60% of the variance, in addition, 3 of the 16 items saturated below 0.70 in their predicted dimension (Table 4).

The overall results of the CFA (GFI 0.957; RMSEA 0.069; CFI 0.973) for the second model assessed (PASTAS-M10) which correspond to the factor structure of the previous model without items 2, 4, 8, 9, 11 and 16 reveal that this measurement model is better than the previous model and that it has an optimal fit to the data (Table 3). The factors in this model explain approximately 61% of the variance. On the other hand, only two of the items saturate below 0.70 in their expected dimension (Table 4). Furthermore, in both models, moderate intercorrelations between the factors were observed, providing evidence of an adequate discriminant validity between them.

The factors obtained from the CFA, in both models, reach internal consistency values above 0.85, evidencing an adequate internal consistency (Table 5).

### 3.3. Confirmatory Factor Analyses for Both Samples

In both the sample of female and male, all the variables show values of skew of ±1.50 and ±2.50 for kurtosis, however, the Mardia multivariate index above the value 70 indicates absence of multivariate normality [36].

As shown in Table 6, in the female sample, the CFA of 10 items grouped into two factors is acceptable (GFI 0.912 and RMSEA 0.104).

Furthermore, in the male sample, results from the confirmatory factor analysis (Table 6) indicate that a two-factor measurement model is optimal (GFI 0.949 and RMSEA 0.068).

The results shown in Table 7, in both samples, most of the items saturated above 0.70 in their predicted dimension. Furthermore, moderate intercorrelations between the factors were observed, providing evidence of an adequate discriminant validity between them.

### 3.4. Invariance of the Factor Structure between Female and Male

The fit indices shown in Table 8 allow for the acceptance of the equivalence of the basic measurement models between the two samples. Although the Chi-squared value exceeds that required to accept the invariance hypothesis, the indices GFI = 0.932, CFI = 0.959, RMSEA = 0.087 and AIC = 24646.080 contradict this conclusion, which allows us to accept the unconstrained model.

By adding restrictions on the base model to the factor loadings, we characterize the metric invariance. The values shown in Table 8 allow us to accept this level of invariance. The general fit index (GFI 0.927) and the mean square error of approximation (RMSEA 0.084) continue to provide convergent information in the sense of metric invariance. In addition, the Akaike Information Criterion (AIC 24645.806) and the Bentler Comparative Index (CFI 0.957) do not show great variations with respect to the previous model. Based on the criteria for the assessment of nested models (Cheung and Rensvold, 2002) [37], which suggests that if the difference of the CFI of both nested models decreases by 0.01 or less, the restricted model is considered to be good and, therefore, the fulfillment of the factorial invariance; the difference between the obtained CFIs allows us to accept the metric invariance model. We can conclude so far that the factor loadings are equivalent in the two samples.

Once the metric invariance between the samples has been demonstrated, we proceed to assess the equivalence between intercepts (strong factorial invariance). The indices (Table 8) show an optimal fit of this model, both independently assessed and analyzed with respect to its nesting with the metric invariance model. The difference between the Bentler comparative indices is one-thousandth; the overall fit index is 0.944 and the root mean square error of approximation is 0.080. Accepting the strong invariance, the two models are equivalent with respect to the factor coefficients and the intercepts.

The factors obtained from the CFAs reached, in both samples (female and male), internal consistency values above 0.85 (Table 9).

### 3.5. Contrasts of the Factor Means between Female and Male Participants

Once the factorial invariance was verified, the differences between the factor means of the two groups were estimated taking the sample of men as reference, setting the value of the means for that sample to 0 and freely estimating the value of the means for the sample of female. The constraints on the regression coefficients and intercepts, required for the contrasts between the means, were automatically performed using the lavaan package. The results of the comparisons indicated that the mean of the non-weight factor was significantly lower (−0.509, *p* < 0.01) in male than in female participants and without differences in the weight factor (−0.064, *p* > 0.05).

## 4. Discussion

The main goal of the present research was to examine the factor structure of the Physical Appearance State and Trait Anxiety Scale (PASTAS-M) questionnaire and the measurement of its factorial invariance in Mexican female and male preadolescents. The following conclusions can be drawn from our results:

(1) The confirmatory factor analyses performed on the total sample support the two-factor structure (weight and no-weight), where the factors thus obtained present adequate standardized factor saturations, which in general, correspond to the proposed structure of the questionnaire developed by Reed et al. (1991), notwithstanding the need to eliminate six of the items. In addition, the positive and statistically significant intercorrelations among the factors show that as anxiety increases in one factor, it also increases in the other.

(2) Confirmatory Factor Analyses: the samples of both female and male preadolescents, indicated that the fit of the data to the theoretical model of 10 items grouped in two factors is at least acceptable. In addition, the obtained factors present adequate standardized factor saturations.

(3) The factors in both samples showed, despite the reduced number of items, an optimal internal consistency.

(4) On the whole, results from the factorial invariance analyses in both samples indicate high congruency among pairs of factors, providing evidence of the cross-validation of the instrument and stability of the structure.

(5) Between groups comparisons reflected significant differences in the non-weight factor (Lips, Hands, Forehead, Neck and Chin), where women show a higher level of anxiety. 

This supports the idea of Sailema et al. (2017) [38] who state that females are more concerned about their physical appearance, becoming more sensitive to physical changes in the ages between 12 and 15 years; however, physical self-concept is related to age since at an early age physical appearance is not considered relevant.

## 5. Conclusions

In conclusion, the analyses of the psychometric properties of the PASTAS -M questionnaire shows that a two-factor structure, according to the established psychometric requirements, is viable, adequate and invariant between pre-adolescent female and male. The two-factor structure shows adequate fit and validity indicators.

## 6. Limitations

The present study has four limitations, however. First, all the participants are students, which poses a threat to the generalizability of the findings. Extending the research to teenagers who are not enrolled in school an area of opportunity for future studies. Second, the instrument used is a self-report measure which may be affected by social desirability bias.

The third limitation is related to the fact that there were various simultaneous adaptations made to the original version of the questionnaire (i.e., translation, change in the response scale and applying it on a computer). As these changes were made at the same time, we were unable to detect whether any one of them could have affected the reliability and validity of the original questionnaire.

The fourth limitation has to do with the sample selection method, which, as it is not probabilistic, introduces the risk of a statistical bias in the results, so it is recommended to take the results with caution.

Likewise, it is necessary to assess whether the questionnaire predicts body dissatisfaction and problems related to eating behaviors.

## 7. Practical Applications

Reducing anxiety caused by one’s own body composition undoubtedly contributes to the quality of life and health of the human being, hence the need to have valid and reliable instruments for its measurement. Therefore, the present study analyzes the psychometric properties proposed by Reed et al. (1991) [20] for the Physical Appearance State and Trait Anxiety Scale (PASTAS-M) questionnaire. This study also serves as a premise for future research on the study of instruments for measuring anxiety related to physical appearance in populations with different personal and cultural factors. Finally, this instrument will be very useful for application in different areas of research, such as, for example, descriptive or intervention studies.

## Figures and Tables

**Table 1:** Number of participants by sex and school grade.

		Sex		
Grade		Female	Male	Total
5	Frequency	150	148	298
	% of total	24.8%	24.5%	49.3%
6	Frequency	135	171	306
	% of total	22.3%	28.4%	50.7%
Total	Frequency	285	319	604
	% of total	47.1%	52.9%	100%

**Table 2:** Skew and kurtosis values of the PASTAS-M questionnaire items. Total sample.

Item	M	SD	SK	KU
Item 1	2.88	2.53	0.79	0.18
Item 2	2.64	2.55	1.02	0.49
Item 3	2.48	2.70	1.12	0.64
Item 4	2.60	2.50	1.00	0.54
Item 5	3.16	2.76	0.71	−0.27
Item 6	2.89	2.70	0.91	0.17
Item 7	2.88	2.65	0.82	−0.08
Item 8	2.96	2.67	0.82	0.01
Item 9	1.84	2.49	1.68	2.51
Item 10	1.85	2.44	1.61	2.35
Item 11	1.78	2.36	1.54	2.14
Item 12	2.04	2.45	1.35	1.44
Item 13	1.83	2.27	1.52	2.27
Item 14	1.87	2.35	1.42	1.74
Item 15	1.69	2.34	1.64	2.40
Item 16	2.46	2.79	1.20	0.67
Mardia multivariate index			89.47	631.34

Note: M = mean, SD = standard deviation, SK = skew; KU = Kurtosis.

**Table 3:** Fit indices for models PASTAS-M16 and PASTAS-M10.

	Absolute Indices			Incremental Indices			Parsimony Indices	
Model	χ^2^	GFI	RMSEA	AGFI	TLI	CFI	CMIN/DF	AIC
PASTAS-M16	513.274 *	0.901	0.081	0.870	0.928	0.939	4.983	39,124.116
PASTAS-M10	131.376 *	0.957	0.069	0.930	0.965	0.973	3.862	24,727.540

Note: * *p* < 0.05.

**Table 4:** Standardized solutions of the confirmatory factor analysis for PASTAS-M16 and PASTAS-M10 models.

Item	PASTAS-M16		PASTAS-M10	
	F1	F2	F1	F2
Factor loadings				
Item 1	0.65		0.64	
Item 2	0.69		-	
Item 3	0.69		0.67	
Item 4	0.79		-	
Item 5	0.77		0.77	
Item 6	0.81		0.82	
Item 7	0.83		0.83	
Item 8	0.74		-	
Item 9		0.71		-
Item 10		0.79		0.78
Item 11		0.81		-
Item 12		0.82		0.80
Item 13		0.81		0.82
Item 14		0.85		0.85
Item 15		0.80		0.81
Item 16		0.75		-
Factor correlations				
F1	-		-	
F2	0.81	-	0.81	-

Note: F1 = weight factor; F2 = non-weight factor.

**Table 5:** Omega and alpha coefficients for the factors from PASTAS-M16 and PASTAS-M10 models.

	PASTAS-M16		PASTAS-M10	
Factor	Ω		Ω	
Weight	0.910	0.909	0.864	0.861
Non-Weight	0.930	0.930	0.905	0.905

**Table 6:** Fit indices PASTAS- M10 for both samples.

	Absolute Indices			Incremental Indices			Parsimony Indices	
Model	χ^2^	GFI	RMSEA	AGFI	TLI	CFI	CMIN/DF	AIC
Female	138.474 *	0.912	0.104	0.858	0.912	0.933	4.080	11,798.118
Male	83.439 *	0.949	0.068	0.918	0.970	0.977	2.454	12,847.963

Note: * *p* < 0.05.

**Table 7:** Standardized solutions for the confirmatory factor analyses for both samples.

Item	Female		Male	
	F1	F2	F1	F2
Factor loadings				
Item 1	0.63		0.64	
Item 3	0.61		0.72	
Item 5	0.79		0.75	
Item 6	0.79		0.84	
Item 7	0.83		0.84	
Item 10		0.69		0.84
Item 12		0.70		0.85
Item 13		0.78		0.86
Item 14		0.87		0.84
Item 15		0.78		0.83
Factor correlations				
F1	-		-	
F2	0.81	-	0.81	-

Note: F1 = weight factor; F2 = non-weight factor.

**Table 8:** Goodness-of-fit indices of each of the models in which factorial invariance was assessed.

Model	Fit Indices						
	χ^2^	gl	GFI	NFI	CFI	RMSEA	AIC
Unconstrained model	221.914 *	68	0.932	0.942	0.959	0.087	24,646.080
Metric invariance	237.640 *	76	0.927	0.949	0.957	0.084	24,645.806
Strong factorial invariance	248.207 *	84	0.944	0.936	0.956	0.080	24,680.374

Note: * *p* < 0.05.

**Table 9:** Coefficients omega and alpha for the obtained factors.

	Female		Male	
Factor	Ω	α	Ω	α
Weight	0.872	0.871	0.857	0.851
Non-Weight	0.871	0.872	0.924	0.924

Note: Ω = Omega; α = Alpha.

## Data Availability

Data available upon request from correspondence author.
